# Measuring and explaining changing patterns of inequality in institutional deliveries between urban and rural women in Ghana: a decomposition analysis

**DOI:** 10.1186/s12939-019-1025-z

**Published:** 2019-08-09

**Authors:** Eugenia Amporfu, Karen A. Grépin

**Affiliations:** 1 Department of Economics, Kwame Nkrumah University of Science and Technology, Kumasi, Ghana; 2 Department of Health Sciences, Wilfrid Laurier University, Waterloo, Ontario Canada

**Keywords:** Oaxaca decomposition, Institutional delivery, Inequality, Poverty

## Abstract

**Background:**

Despite recent progress in improving access to maternal health services, the utilization of these services remains inequitable among women in developing countries, and rural women are particularly disadvantaged. This study sought to measure i) disparities in the rates of institutional births between rural and urban women in Ghana, ii) the extent to which existing disparities are due to differences in the distribution of the determinants of institutional delivery between rural and urban women, and iii) the extent to which existing disparities are due to discrimination in resource availability.

**Methods:**

Using Demographic and Health Survey data from 2003, 2008, and 2014, this study decomposed inequalities in institutional delivery rates among urban and rural Ghanaian woman using the Oaxaca, the Blinder, and related decompositions for non-linear models. The determinants of the observed inequalities were also analyzed.

**Results:**

Institutional delivery rates in urban areas exceeded those of rural areas by 32.4 percentage points due to differences in distribution of the determinants of institutional delivery between the two areas. The main determinants driving the observed disparities were wealth, which contributed to about 16.1% of the gap, followed by education level, and number of antenatal visits.

**Conclusion:**

Relative to urban women, rural women have lower rates of institutional deliveries due primarily to lower levels of wealth, which results in financial barriers in accessing maternal health services. Economic empowerment of rural women is crucial in order to close the gap in institutional delivery between urban and rural women.

## Background

Despite progress in reducing maternal mortality worldwide, pregnancy and childbirth remain a major cause of death for women in low- and middle-income countries (LMICs). In 2015 alone, it was estimated that over 300,000 women globally died as a result of pregnancy or childbirth, almost two thirds of whom resided in Africa [3]. Given that the majority of these deaths are preventable with timely access to maternal healthcare, institutional delivery has been widely promoted as a key strategy for reducing maternal mortality [5, 18, 25]. Indeed, healthcare institutions have the capacity to provide emergency obstetric care and ensure that births are attended by a skilled health professional - both of which are believed to be critical to the reduction of maternal mortality [5, 18, 25, 27].

While institutional delivery rates have been increasing worldwide, substantial disparities persist within countries. In additional to sociodemographic factors, such as wealth status and education [8, 22], studies have identified urbanicity as a significant determinant of institutional delivery and have observed higher rates of institutional births in urban areas compared to rural areas in a number of countries, holding all other factors constant [19, 27]. In Ghana, a lower-middle income country in West Africa which had a maternal mortality ratio (MMR) of 310 deaths per 100,000 live births in 2017, just 59.0% of rural births occurred in an institutional setting, compared to 90.2% of urban births [14]. Given that almost half of Ghana’s population (49.1%) resides rurally [12], further improvements in national maternal health outcomes cannot be achieved without increasing rates of institutional delivery in rural areas. As such, a greater understanding of the underlying factors contributing to urban-rural disparities in institutional delivery is necessary in order to develop effective intervention strategies to close this gap.

### Previous research on institutional delivery and Urbanicity

Past studies examining differences in institutional deliveries between urban and rural women have examined urbanicity as a determinant of institutional delivery and have generally found higher rates in urban areas, holding all the other determining factors constant. For example, Stephenson, et al. [27] showed that institutional deliveries in urban areas exceed those in rural areas in Tanzania, Malawi, and Ghana, while Mehari [19] found similar results in Ethiopia. These studies, however, did not attempt to explain the observed differences in rates of institutional deliveries between rural and urban women. Other studies (e.g., [7, 10, 28]) have examined determinants of institutional deliveries in rural areas without comparing to urban areas.

Afful-Mensah et al. [2] used data from Ghana’s Demographic Health Survey (DHS) to explore the difference between rural and urban institutional delivery rates in Ghana using two separate regressions. The coefficients from each regression were then compared to determine which factors had a significant impact on rural versus urban institutional deliveries. Such an approach has two problems. First, it is unknown whether changing the significant determinants identified in the rural regression could close the gap in institutional delivery between rural and urban areas. Second, the study was not able to link differences in the determinants of institutional delivery observed in descriptive statistics with the results of the regressions to highlight the most important determinants for closing the gap. The authors were therefore not able to provide potential explanations for the gap.

The following paper aims to explore the causes of the persistently lower institutional delivery rates in rural compared to urban Ghana using the Oaxaca and related decompositions to quantify the gap in institutional delivery into two parts; that explained by differences in the levels of the determinants of institutional delivery between urban and rural-dwellers, and that explained by differences in the effect of the determinants on institutional delivery between urban and rural-dwellers. To our knowledge, no study has previously attempted to distinguish between the two explanations for the urban-rural disparity in the utilization of maternal health services in any international context.

## Methods

### Data source

This study analyzed data from the three most recent rounds of the Ghana Demographic and Health Survey (DHS): a large, nationally representative household survey that collected information on fertility and family planning, infant and child mortality, maternal and child health, nutrition, malaria, HIV/AIDs, and a number of other household characteristics using questionnaires administered by trained interviewers. Additional details about the survey methodology and sampling procedures can be found elsewhere [13, 15].

### Study population

For the purposes of this study, our sample was restricted to women of reproductive age (15–49 years), and focused on their most recent births during the 5 years preceding each of the 2003, 2008, and 2014 DHS surveys. Thus, the final sample spanned the years 1999–2014 and included 13,802 births; of which 5672 (41.10%) occurred in urban areas and 8130 (58.90%) occurred in rural areas.

### Measures

#### Dependent variable

Institutional delivery was the primary outcome of interest and was assessed using self-reported data on the location of delivery of all births that occurred within 5 years of the dates of the surveys. For analyses, it was considered a binary variable and was classified as one if a woman delivered in a public or private healthcare institution and zero otherwise.

#### Independent variable

Area of residency was the key independent variable and was a binary; classified as urban or rural based on the definition used in the 2010 Population and Housing Census. Under this definition, communities were considered urban if they had a population of 5000 persons or greater and rural if they had a population of fewer than 5000 persons [12].

#### Explanatory variables

Additional data were extracted from the DHS surveys and were treated as covariates in all analyses. These included factors that are known to be associated with institutional delivery and/or area of residency, such as wealth quintile (ordinal categorical variable), education level (ordinal categorical variable with categories ‘no education’, ‘primary education’, ‘secondary education’, and ‘tertiary education’), parity (continuous variable representing the number of children born to a woman) and distance from a health facility (binary variable classified as one if the woman considered distance to be a barrier to accessing care and zero otherwise), as well as more general sociodemographic factors such as age (ordinal categorical variable with categories ‘15–19’, ‘20–24’, ‘25–29’, ‘30–34’, ‘35–39’, ‘40–44’, and ‘45–49’), ethnicity (nominal categorical variable with categories ‘Ga’, ‘Akan’, ‘Ewe’, and ‘a tribe from the three northern regions’), and religion (nominal categorical variable with categories ‘Christian’, ‘Muslim’, ‘Traditionalist’, ‘other’, and ‘no religion’) and pregnancy-related factors such as pregnancy complications (dummy variable classified as one if a woman had complications and zero otherwise), antenatal care visits attended (continuous variable), contraceptive use (binary variable classified as one if a woman practiced family planning and zero otherwise), and birth year (categorical variable with categories for each year from 1999 to 2014). A variable denoting the year of the survey (categorical variable with response options ‘2003’, ‘2008’, and ‘2014’) was also included.

Given that the Ghanaian government has previously introduced reforms in an attempt to increase maternal healthcare utilization and improve outcomes, we also chose to include a categorical variable denoting the reform period in which the birth occurred for all analyses, so as to control for the effects these may have had on service usage. This variable took on a value of one for births that took place outside of the reform periods, two for the first reform period, which was characterised by the provision of free maternal care in public facilities and was in place from 2003 until 2007, when it was integrated into the already functional National Health Insurance Scheme (NHIS) [16], and three for the second reform period, which was introduced in 2008 and provides free NHIS enrolment for all pregnant women, who are enrolled automatically at their first antenatal care visit for a period ending 3 months after their delivery. Under this scheme, all maternity care is covered free of charge in all healthcare facilities, including antenatal care, delivery (vaginal or caesarean), and emergency care [16].

#### Statistical analyses

Statistical analyses were conducted using STATA. Descriptive statistics including means for continuous variables and percentages for categorical variables were calculated to describe the characteristics of the study sample by area of residency. The Oaxaca, Blinder, Reimers, and Cotton decomposition methods for non-linear models were used to explain the gap in rates of institutional delivery between urban and rural women. According to the Oaxaca decomposition theory, differences in the mean of an outcome for two groups can be explained by differences in the level or distribution of the determinants of the outcome (explained component), differences in the impact of these determinants on the outcome (unexplained component), and/or the interaction of the two [21].

Assume a regression model that links *Y*, the outcome variable, to a set of covariates, *X* with a vector of coefficients, *β*.

*Y*^*j*^ = *β*^*j*^*X*^*j*^ where *j* = rural, urban.

The difference between $\overline{Y}$ ^*urban*^ and $\overline{Y}$ ^*rural*^ (where $\overline{Y}$ represents average) can be written in two ways:

1. Oaxaca decomposition

$$ {\overline{Y}}^{urban}-{\overline{Y}}^{rural}=\Delta  \overline{X}{\beta}^{urban}+\Delta  \beta {\overline{X}}^{rural}+\Delta  \overline{X}\Delta  \beta $$

Where $\Delta  \overline{X}$ is the difference between $\overline{X}$ ^*urban*^ and $\overline{X}$ ^*rural*^ and the similarly for *∆β*.

(2) Blinder decomposition

$$ {\overline{Y}}^{urban}-{\overline{Y}}^{rural}=\Delta  \overline{X}{\beta}^{rural}+\Delta  \beta {\overline{X}}^{urban}+\Delta  \overline{X}\Delta  \beta $$

Equations (1) and (2) are equivalent and describe the decomposition of the difference between the outcomes of the groups. The three terms on the right-hand side represent the three components of the difference between the outcomes. The first two components (the explained and unexplained components) are the average differences between the *Xs* of the urban and rural women and that of the *βs,* with each multiplied by weights. Typically, the first component is weighted by coefficients while the second component is weighted by covariates.

The Oaxaca [20] decomposition (1) uses the high group (urban women in this study) as the reference group, weighting differences in characteristics by the coefficients of urban women and differences in coefficients by the covariates of rural women. The Blinder (1973) decomposition (2) does the opposite, using the low group as the reference group (rural women in this study), and weighting differences in characteristics by the coefficients of the rural women and differences in coefficients by the covariates of the urban women. Therefore, the Oaxaca decomposition assumes that the outcome of the high group is in accordance with their characteristics, and that of the low group is due to discrimination against them while the Blinder decomposition assumes that the outcome of the low group is in accordance with their characteristics and that of the high group is the result of societal favoritism. Thus, while the unexplained component of the Oaxaca decomposition focuses on discrimination against the low group, the Blinder’s focuses on favoritism of the high group [21].

Other studies propose using the weighted averages of the two groups as weights. Hence, the weight of the differences in covariates is equal to the weighted mean of the coefficients of the urban and rural groups. According to Reimers [24], the weighted mean should be computed as 0.5 (equal weights for the two groups), while Cotton [6] believes it should be the proportions of the two groups in the sample. Because the outcome of the decomposition is sensitive to the weighting method used [17], this study ran a different decomposition for each method: Oaxaca, Blinder, Reimers, and Cotton. Regressions were performed for urban and rural women separately and then the estimated coefficients and covariates were used to compute the decompositions. Consistent results using the different weights were thought to represent robustness of the study outcome.

The equation for the decomposition analysis conducted in this study is specified as follows:

(3) Decomposition Equation

$$ {y}_i^j={\beta}_i^j+{\beta}_2^j{X}_{2i}^j+{\beta}_3^j{X}_{3i}^j+{\beta}_4^j{X}_{4i}^j+{\beta}_5^j{X}_{5i}^j+{\beta}_6^j{X}_{6i}^j+{\beta}_7^j{X}_{7i}^j+{\beta}_8^j{X}_{8i}^j+{\beta}_9^j{X}_{9i}^j+{\beta}_{10}^j{X}_{10i}^j+{\beta}_{11}^j{X}_{11i}^j+{\beta}_{12}^j{X}_{12i}^j+{e}_i^j $$

*j = U, R U* = urban, and *R* = rural.

Where *y* represents the outcome variable, institutional delivery, *X*_*2*_ represents age, *X*_*3*_ represents education, *X*_*4*_ represents household wealth quintile, *X*_*5*_ represents parity, *X*_*6*_ represents pregnancy complications, *X*_*7*_ represents distance from a healthcare facility as a perceived barrier, *X*_*8*_ represents the maternal care reform period, *X*_*9*_ represents the number of antenatal visits the mother attended, *X*_*10*_ represents ethnicity, *X*_*11*_ represents religion, and *X*_*12*_ represents survey year. Four decompositions were estimated: one pooling the data from the three survey years used in the study, and one for each individual survey year.

The Heckman Selection Model approach was used to account for possible selection bias wherein only women who had already given birth could be selected for inclusion. The Heckman approach is a two-stage procedure involving the estimation of the selection equation using the larger sample by probit and then computing the inverse mills ratio variable using the predicted outcome in the first stage, and the estimation of the equation of interest with the inclusion of the inverse Mills ratio variable in the second stage. In this study, the dependent variable of the selection equation was a previous delivery and the independent variables were individual-level characteristics including the mother’s age, education level, wealth status, religion, ethnicity, marital status, location of residency, and use of contraceptives. The Mills ratio computed was then added to the response eq. (3) for estimating the decompositions.

Overall decompositions were computed to determine the contribution of the explained component, along with that of the unexplained component plus the contribution of the interaction to the gap in the outcome. Additionally, detailed decompositions were computed to determine the contribution of each independent variable to the explained and unexplained decompositions, as described by Kaiser [17]. Following the methods of Kaiser [17], the detailed decomposition analyses focused only on the explained decomposition.

## Results

### Characteristics of the sample

The sociodemographic and pregnancy-related characteristics of the sample are presented in Table 1. Almost two thirds of women (65.5%) resided rurally, while just over one third (34.5%) were urban. A lower proportion of rural women had a formal education, with 48.6% reporting no education, compared to 21.37% of urban women, and the majority of rural women (76.64%) belonged to the poorest two wealth quintiles, while the majority of urban women (65.80%) belonged to the richest two. Rural women primarily belonged to ‘Other’ tribes (48.81%), followed by Akans (34.87%), while urban women were mostly Akans (48.98%) followed by ‘Other’ tribes (32.15%). Christian was the predominant religion for both rural and urban women, practiced by 66.35 and 71.86% of women, respectively, followed by Muslim. The proportion of women who were married or living with their significant other was very similar regardless of location, however slightly more urban women had never been married than rural women.

**Table 1:** Sociodemographic and pregnancy-related characteristics of the sample

Characteristic	Full Sample		2003		2008		2014	
	Rural	Urban	Rural	Urban	Rural	Urban	Rural	Urban
Proportion of the Sample	65.5	34.5	72.9	27.1	66.6	33.4	60.2	39.8
Sociodemographic Characteristics								
*Age (%)*								
15–19	3.81	3.27	3.39	3.45	3.66	4.60	4.15	2.56
20–24	19.11	16.28	18.17	18.70	20.88	16.70	18.50	15.10
25–29	26.49	26.87	26.06	27.13	27.86	26.90	24.55	26.66
30–34	20.91	25.67	21.46	24.83	18.42	23.60	22.01	26.88
35–39	17.36	17.98	17.67	15.72	16.72	18.70	17.66	18.60
40–44	9.08	7.41	8.78	6.71	8.79	6.70	9.52	8.19
45–49	3.84	2.51	4.46	3.45	3.66	2.80	3.62	2.01
Education (%)								
No education	48.60	21.37	56.09	24.26	47.34	18.90	43.67	21.26
Primary education	22.67	19.69	20.85	22.91	24.75	22.90	22.91	16.98
Secondary education	28.05	52.68	22.78	49.76	27.36	52.80	32.37	53.88
Tertiary education	0.68	6.26	0.29	3.07	0.55	5.40	1.05	7.98
Ethnicity (%)								
Akan	34.87	48.98	36.48	54.97	34.18	50.21	34.22	46.01
Ga/Dangme	4.52	6.96	6.51	8.82	3.93	6.90	3.44	6.18
Ewe	11.79	11.91	12.32	10.87	12.77	12.74	10.88	12.02
Other	48.81	32.15	44.69	25.33	49.12	30.15	51.46	35.79
Wealth Quintile (%)								
1 (Poorest)	46.80	5.67	44.84	2.78	47.89	1.90	47.66	8.49
2	29.84	7.58	29.13	4.12	29.07	7.70	30.90	8.96
3	16.23	20.96	18.53	15.63	12.90	22.90	15.79	22.35
4	5.88	32.59	5.53	36.82	7.58	35.10	5.14	29.91
5 (Wealthiest)	1.25	33.21	1.96	40.65	1.66	32.40	0.51	30.29
Region of Residence (%)								
Western	14.43	8.45	9.39	8.53	8.84	9.40	11.10	8.06
Central	8.71	8.01	6.82	4.79	8.23	6.30	10.31	10.15
Greater Accra	2.31	20.15	2.68	25.31	1.76	24.40	2.37	16.04
Volta	8.72	6.17	8.10	4.51	9.34	5.90	8.95	7.00
Eastern	9.31	7.99	8.89	6.42	9.34	7.50	9.60	8.75
Ashanti	10.05	17.68	12.53	21.76	12.45	19.10	6.78	15.32
Brong Ahafo	10.42	11.47	11.25	13.81	8.73	9.20	10.82	11.52
Northern	18.62	10.34	18.71	9.49	18.72	10.60	18.42	10.67
Upper East	10.00	5.36	9.75	1.73	9.79	3.20	10.37	7.85
Upper West	11.87	4.37	11.89	3.64	12.80	4.40	11.27	4.65
Marital Status (%)								
Married/Co-habiting	87.35	87.06	92.25	88.97	92.12	87.70	87.49	85.75
Formerly married	5.57	6.17	5.93	6.62	4.62	5.80	6.33	6.36
Never married	3.92	6.77	1.82	4.41	3.26	6.50	6.19	7.89
Religion (%)								
Christian	66.35	71.86	61.30	77.37	64.41	69.40	71.38	70.48
Muslim	18.16	24.66	20.71	20.81	16.82	26.70	17.09	25.55
Traditional	8.06	0.99	7.78	0.19	12.15	1.40	5.93	1.15
No religion	7.33	2.42	10.07	1.63	6.43	2.20	0.00	0.00
Other	0.10	0.07	0.14	0.00	0.20	0.30	5.59	2.82
Distance to Healthcare Facility (%)								
Major barrier to accessing care	45.34	17.55	53.16	15.34	41.62	17.40	41.07	18.52
Not a barrier/small barrier to accessing care	54.53	82.45	46.84	79.87	58.03	82.60	58.93	81.48
Pregnancy Characteristics								
Number of antenatal visits attended (mean)	7.13	8.89	7.78	12.10	7.69	9.47	6.35	7.28
Attended 4+ antenatal visits (%)	74.23	91.03	64.74	88.86	73.19	90.43	82.36	92.33
Experienced pregnancy complications (%)	67.88	79.27	54.43	68.24	63.80	76.23	79.86	85.53
Delivered in an institutional setting (%)	43.10	84.69	27.70	78.33	39.16	82.80	56.50	88.10
Parity (mean)	4.05	3.13	4.17	3.24	4.00	3.09	4.00	3.12
Total Observations	8259	4343	2801	1043	1992	1000	3540	2344

In terms of pregnancy characteristics, both the number of antenatal visits attended and the proportion of women who delivered in an institution were consistently higher among urban populations compared to rural. Indeed, more than 90% of urban mothers made at least the minimum of four antenatal visits versus 74% of rural mothers, although when disaggregating by year, the results show an upward trend in the proportion of women attending four or more antenatal visits for both urban and rural women, with a higher increase observed for rural women. Overall, institutional delivery rates among urban women exceeded those of rural women by 41.6 percentage points. Across the survey years, institutional deliveries increased faster for rural women compared to urban women, resulting in a narrowing of the gap from 50.6 percentage points in 2003 to 31.6 percentage points in 2014. A higher proportion of urban women reported experiencing complications during pregnancy (79.27% versus 67.88% of rural women), and rural women had, on average, one child more than urban women. Almost half of rural women (45.34%) felt that distance to a healthcare facility was a major barrier in accessing care, compared to just 17.55% of urban women.

### Decomposition estimates

The results of the decomposition analyses are demonstrated in Table 2. Minimal differences in the results were observed when antenatal visits were included in the models compared to when they were not (see Appendix), indicating that post-treatment bias is unlikely. The results of the equations that included antenatal visits were thus prioritized, as doing so avoids endogenization of variables in the equations correlated with antenatal visits.

**Table 2:** Decomposition estimates

	a: Combined Data		b: 2003 Data		c: 2008 Data		d: 2014 Data	
	Coefficient	Percentage	Coefficient	Percentage	Coefficient	Percentage	Coefficient	Percentage
	Oaxaca Decomposition: Weight = 1							
Explained Component	0.234^a^	72.3	0.306^a^	65.3	0.300^a^	75.3	0.192^a^	68.9
Unexplained Component	0.136^a^	42	0.159^a^	34	0.204^a^	51.1	0.122^a^	43.7
Interaction	−0.046	−14.3	0.003	0.7	−0.105^a^	−26.4	− 0.035	−12.6
Total	0.324^a^	100	0.468^a^	100	0.399^a^	100	0.278	100
	Blinder Decomposition: Weight = 0							
Explained Component	0.189^a^	58.1	0.309^a^	66	0.195^a^	48.9	0.156^a^	56.2
Unexplained Component	0.089^a^	27.6	0.162^a^	34.7	0.099^a^	24.7	0.087^a^	31.1
Interaction	0.046	14.3	−0.003	−0.7	0.105	26.4	0.035	12.6
Total	0.324^a^	100	0.468^a^	100	0.399^a^	100	0.278	100
Explained Component	Reimers Decomposition: Weight = 0.5							
	0.214^a^	66.3	0.319^a^	68.2	0.254^a^	63.6	0.177^a^	63.7
Advantage	0.038^a^	11.8	0.071^a^	15.2	0.043^a^	10.7	0.035^a^	12.4
Disadvantage	0.071^a^	21.9	0.077^a^	16.6	0.102^a^	25.6	0.067^a^	23.9
Total	0.324^a^	100	0.468^a^	100	0.399^a^	100	0.278	100
	Cotton Decomposition							
	Weight = 0.60		Weight = 0.68		Weight = 0.66		Weight = 0.57	
Explained Component	0.209^a^	64.7	0.318^a^	67.95	0.237^a^	59.4	0.174^a^	62
Advantage	0.029^a^	9.1	0.043^a^	9.1	0.029^a^	7.3	0.028^a^	10
Disadvantage	0.085^a^	26.2	0.108^a^	22.9	0.133^a^	33.3	0.077^a^	28
Total	0.324^a^	100	0.468^a^	100	0.399^a^	100	0.278^a^	100

^a^Significant at the 5% level

Table 2a describes the decomposition estimates of each method using data pooled across all survey years. The average total difference in predicted institutional delivery rates between the urban and rural groups was 0.324, indicating that the institutional delivery rate of urban women exceeded that of rural by 32.4 percentage points on average, regardless of the type of decomposition used. The proportion of this gap due to explained and unexplained components varied depending on the weighting used, however positive coefficients were consistently observed across all decomposition methods, indicating that the components contribute to a widening of the gap, rather than a narrowing. Furthermore, regardless of the decomposition type, the explained component always exceeded the unexplained.

As discussed previously, the Oaxaca decomposition assumes that the unexplained component is due to discrimination against rural women, while the Blinder assumes it is due to favoritism toward urban women. In Table 2a, the unexplained component is approximately 42% when using the Oaxaca decomposition, compared to 28% when using the Blinder decomposition, suggesting that discrimination against rural women contributes more to the gap in institutional delivery than favoritism of urban women. These results are confirmed by the Cotton and Reimer decompositions, which are able to show how both discrimination and favoritism contribute to the gap. Indeed, using the Cotton decomposition, only 9.1% of the gap is explained by advantage to urban women, while 26.2% is explained by disadvantage to rural women. Similarly, when using the Reimers decomposition, 11.8% of the gap is explained by advantage to urban women, compared to 21.9% explained by disadvantage to rural women.

The results from the three individual surveys as demonstrated in Table 2b-d reveal a gradual reduction in the gap in institutional delivery rates between urban and rural women over time: from about 47 percentage points in 2003 to about 28 percentage points in 2014 in the Oaxaca decomposition. Across all three surveys, the explained component formed the largest percentage of the gap, ranging from 65.3 to 75.3% for the Oaxaca decomposition and 48.9 to 66.0% for the Blinder decomposition. This suggests that a greater proportion of the gap in institutional delivery rates between rural and urban women can be explained by differences in the determinants of institutional delivery among these groups. Additionally, the explained component increased over the years. Improving the determinants of institutional delivery for rural women to the level of those of urban women could therefore close the gap in institutional delivery by approximately 48.9 to 75.3%.

Under the Oaxaca decomposition, the unexplained component, representing the contribution of discrimination against rural women to the gap in institutional delivery, increased over time: from about 34% in 2003 to 43.8% in 2014. However, under the Blinder decomposition the unexplained component, representing the contribution of favoritism towards urban women to the gap in institutional delivery, decreased over time: from 34.7% in 2003 to 31.1% in 2014. These findings are confirmed by the decrease in the advantage component and the increase in the disadvantage component observed in the Cotton and Reimer’s decompositions. With the exception of the 2008 survey, the interaction component made the smallest contribution to the gap in institutional delivery. The significant change in the unexplained component in 2008 and onward could be due to the maternal care reform in 2008 that integrated the free maternal care program into the NHIS hence expanding the free maternal care to all women, rural or urban. The resulting increase in institutional delivery rates must have been greater in the urban than rural areas due to the disadvantage conditions in rural areas. Such disadvantage could include lack of access to health facilities for the free maternal care.

### Detailed decomposition estimates

The contributions of each of the determinants of institutional delivery to the gap in institutional delivery rate between urban and rural women are reported in Table 3. In general, these results did not vary by weighting method.

**Table 3:** Contribution of the Determinants of Institutional Delivery to the Gap Between Urban and Rural Women

	Pooled Data	2003 Data	2008 Data	2014 Data
	Explained Effect	Explained Effect	Explained Effect	Explained Effect
Component	0.261^a^	0.287^a^	0.309^a^	0.217^a^
Four+ Antenatal Visits	0.019^a^	0.037^a^	0.019^a^	0.018^a^
Pregnancy Complications	0.0002	−0.0001	0.0003	0.0003
Parity	0.023^a^	0.01	0.018	0.027^a^
Age	0.001	−0.002	0.0001	0.002
Education	0.039^a^	0.031^a^	0.043^a^	0.034^a^
Wealth Index	0.161^a^	0.177^a^	0.228^a^	0.114^a^
Region	−0.017^a^	−0.016	− 0.017	−0.014^a^
Ethnicity	0.001	0.018^a^	0.001	0.001
Religion	0.01^a^	0.01	0.013^a^	0.006^a^
Marital Status	0.003	0.0001	0.0002	0.0004
Distance from Health Facility	0.016^a^	0.014	0.008	0.023^a^
Inverse Mills Ratio	−0.002	0.006	−0.008	0.006
Maternal Health Reforms	−0.002	–	–	
Survey Fixed Effect	0.01	0	–	0
Constant			0.235	0.351
Unexplained Effect	0.025^a^	0.181^a^	0.091^a^	0.062^a^

^a^Significant at the 5% level

The results of the pooled data analysis demonstrate that education, wealth, parity, antenatal visits, region of residence, religion, and distance to a health facility all contributed significantly to the inequity in institutional delivery observed between rural and urban women, with wealth differences contributing the most. With the exception of region of residence, which mitigated the inequity, all of these factors widened the gap between the two groups. In examining the results of the individual surveys, antenatal visits, education, and wealth contributed to the widening of the gap regardless of the period, while region of residence contributed to the narrowing of the gap. Across all survey periods, wealth differences contributed the most to the inequity between rural and urban women, followed distantly by education. The proportion of the gap in institutional deliveries due to wealth differences increased from 17.7 percentage points in 2003 to 22.8 percentage points in 2008, but decreased to 11.4 percentage points in 2014, potentially due to the introduction of free NHIS enrollment for pregnant women in 2008. Despite this, wealth remained the dominant contributor to the inequality. Region of residence contributed narrowly to the closing of the gap in all periods, while the remaining variables made marginal contributions to widening it.

## Discussion

There is substantial evidence to suggest that institutional delivery rates in developing countries are higher in urban areas compared to rural [19, 27]. Research examining the factors that are driving this gap, however, has been lacking to date. Given that institutional births have been linked to improved maternal health outcomes including reduced mortality rates, closing the gap is of the upmost importance. This study described the disparities in the rates of institutional delivery between urban and rural woman in Ghana, West Africa, and examined the underlying causes driving this inequality using the Oaxaca and related decompositions. To our knowledge this is the first study to attempt to explain the observed gap in institutional births between urban and rural woman.

### Differences in the levels of the determinants of institutional delivery

Our results demonstrate that over 50% of the observed gap in rates of institutional delivery among urban and rural women in Ghana can be attributed to differences in the distribution of the determinants of institutional delivery between these groups, irrespective of the decomposition type and survey year. In other words, urban women are more likely to give birth in an institutional setting because they are better endowed with the determinants that favour institutional delivery. In particular, we found that wealth, education, parity, antenatal care visits, religion, region, and distance from a health facility were all important contributors to the urban-rural gap in institutional delivery, with all factors except for region acting to widen the disparity. These results are in line with the findings of previous studies, which identified wealth, education, antenatal visits, and parity as important predictors of institutional delivery [8, 22, 26], and are supported by our descriptive analysis, which observed higher levels of education and wealth, a higher proportion of women attending four or more antenatal care visits, a lower proportion of women reporting distance to a health facility to be a barrier in accessing care, and lower parity among urban women compared to rural women.

Across all survey years, wealth differences contributed the most to the gap in institutional delivery. The pooled data in Table 3 shows that wealth contributed about 61.7% (0.161/0.261) to the explained component indicating that rural women deliver in institutions at lower rates primarily due to financial barriers. This is confirmed by the descriptive statistics presented in Table 1, which demonstrate that over 60% of urban women fell into the top two wealth quintiles, compared to less than 10% of rural women. Wealth contributes 16.1% of the gap in institutional delivery between rural and urban women, implying that raising the wealth of rural women to the level of urban women could reduce the gap in institutional delivery by as much as 16.1%. Interestingly, wealth remained the primary driver of the gap in institutional delivery even after the introduction of free NHIS for all pregnant women in 2008, although its contribution did decline. This could be because women had to attend antenatal care visits in order to be enrolled in the program, and a lower proportion of rural women received antenatal care compared to urban women, supporting the finding that wealth status is an important determinant of maternal care utilization in low- and middle income countries [4]. Alternatively, it could suggest that poverty impacts institutional delivery through channels other than just the affordability of care. Poor women may not be able to obtain transportation to a health institution, for example, or may not be able to find childcare. As such, financial accessibility must be considered more broadly.

Along with wealth, differences in education levels between urban and rural women was an important contributor to the gap in institutional delivery, explaining approximately 15% of the inequality. Importantly, the contribution of education did not appear to decrease across survey years, suggesting that urban-rural inequalities in education may not be improving. The positive coefficients imply that urban women have higher levels of education on average compared to rural women. This is confirmed by Table 1 which shows that the proportion of women with secondary and tertiary education was higher among urban women than rural women. In addition, the increase in the proportion of women with tertiary education grew faster over the years in urban areas than rural areas. The reason for such a gap could be because it is easier for women with tertiary education to find jobs in urban areas than rural areas. Given that higher levels of education have positive relationship with institutional delivery [8, 22], encouraging rural women to attain higher education could help close the gap.

A final important determinant of the gap in institutional delivery between urban and rural women was the attendance of antenatal visits, although its contribution to the inequity was found to decrease over time. Descriptive statistics reveal that this decline was likely the result of antenatal care visit attendance increasing more rapidly in rural compared to urban areas over the study periods, however urban women were still more likely to meet the minimum of four recommended visits. The positive relationship between antenatal visits and institutional delivery is well documented, and women who attend more antenatal visits are more likely to deliver in an institution irrespective of region [29].

### Differences in the effect of the determinants of institutional delivery

Along with differences in the distribution of the determinants of institutional delivery between urban and rural areas, the urban-rural gap in institutional delivery can also be attributed to differences in the effect of the determinants on increasing institutional delivery rates in rural compared to urban areas, although to a lesser extent. The positive coefficients of the unexplained component imply that a change in a determinant that increases institutional delivery would lead to smaller improvement in rural areas compared to urban. For example, because the quality of education is worse in rural areas [23], an educated rural woman may not have enough knowledge to value institutional delivery to the same extent as an urban woman with equivalent schooling. Similarly, because rural health facilities are less equipped [11], accessing a quality facility for institutional delivery may be difficult, even for a rich woman. Thus, an increase in wealth would increase the likelihood of institutional delivery for poor urban women more than poor rural women.

The unexplained component of the decomposition, which can also be thought of as favoritism or discrimination that leads to differences in institutional delivery, contributed between 9 and 42% of the observed disparity in institutional delivery rates in urban versus rural Ghana, compared to 58.1–72.3% contributed by the explained component. The wide range of the contribution of unexplained component is due to the different weights used by the various methods. The Blinder method assuming no discrimination but only favouritism of urban women shows a high contribution of favouritism (28%) to the inequality than the Cotton (9%) and Reimer (11.1%) methods which assumed the gap is partly due to favouritism of urban women and partly due to discrimination against rural women. Similarly, the Oaxaca decomposition which assumes discrimination against rural women shows a higher contribution (42%) of the unexplained component to the inequality. Even though the results are sensitive to the weights chosen by each method of decomposition, the results consistently demonstrate that discrimination against rural women contributed more to the observed gap in institutional delivery rates than favoritism towards urban women.

### Policy implications

Given that institutional delivery is crucial in order to improve maternal mortality rates, which are disproportionately high among rural women, efforts must be made to close the urban-rural gap in institutional delivery, particularly by targeting the factors that contribute most. Effective policies to lift rural women out of poverty, for example, must be developed if any significant improvements in institutional delivery rates are to be achieved. These programs should be sensitive to rural circumstances and must address cultural barriers that may prevent women from generating an income. Indeed, the majority of rural women in Ghana work as farmers, and collectively contribute up to 70% of all agricultural labour and produce up to 70% of all food. These women face discrimination in land acquisition, however, preventing them from controlling agricultural outputs and thus earning an income, and this results in continuing poverty [1].

In addition to wealth, programs to increase education levels among rural women must be implemented in order to improve rates of institutional delivery. Education, after all, increases a women’s earning potential and also makes her more knowledgeable about the benefits of giving birth in an institutional setting. Currently, rural children attend school at lower rates than urban children, primarily due to issues of poverty. Indeed, the widespread rural poverty that we observed in this study often requires children participate in household or agricultural labour instead of going to school, or else prevents them from paying school fees, purchasing school equipment, or travelling the, sometimes, long distances to school [9]. Where rural children do attend school, they tend to receive a poorer quality education, due to a lack of resources in rural schools compared to urban. This can lead to disparities in student performance that may prevent a rurally-educated child from qualifying for higher education [23]. In line with this, the literacy rate in rural Ghana was 62.8% in 2010, compared to 84.1% in urban Ghana [12]. Thus, interventions that promote school attendance are necessary. These should involve both increasing the quality of the education provided, as well as increasing assess to education. For example, the free Senior High School introduced in 2017 that waives school fees, providing books and uniforms to students could give more attention to rural schools to ensure access for rural youth especially girls to quality secondary education.

Efforts to increase antenatal visits in rural areas is needed to close the gap in antenatal visits between rural and urban women. The positive relationship between antenatal visits and institutional delivery is well documented thus antenatal visits in rural areas need to rise to the level of urban areas. To achieve this, antenatal services need to be available in rural health facilities. Finally, implementation of maternal programs should be location sensitive. Making free maternal healthcare accessible in rural areas may require frequent use of mobile clinics, as well as increasing the number and quality of rural health facilities. Specifically, extending the Community-based Health Planning and Services (CHPS compound) initiative to rural communities is likely to enhance healthcare utilization including antenatal care visits and institutional deliveries. CHPS compound program could be very effective in increasing antenatal visits because the program is well integrated into the community and so the health education in the program could include educating pregnant women to go for antenatal care and deliver in health facilities.

### Limitations

This study was not without limitation. First, the data analyzed here is relatively outdated; thus, the findings may not accurately reflect the current situation in Ghana with regards to institutional delivery. While more recent survey data does exist, it was not collected using the DHS but rather a different survey instrument, and may therefore not be comparable to earlier data. As such, we chose to limit our analysis solely to DHS data. Second, this study made use of self-reported data, which is vulnerable to recall bias, particularly given that each survey recorded information for the 5 years preceding the survey. Because the survey focused on births, however, which are considered to be big events that are not soon forgotten, we expect the accuracy of the data to be acceptable.

## Conclusions

Using various decompositions, the present study of inequalities in institutional delivery in urban compared to rural Ghana found that the rates of institutional births among urban women exceed those of rural women by approximately 32.46 percentage points. This gap was primarily the result of differences in the distribution of the determinants of institutional delivery between urban and rural women, and wealth disparities were particularly important, contributing around 16.1% of the inequality. Future interventions to promote institutional births in rural Ghana should therefore focus on economic empowerment, and should aim to reduce the existing financial barriers that prevent rural women from accessing health care. Additional research is required in order to identify the policies and programs that may be most effective at lifting rural women out of poverty. The gap is also due to the discrimination against rural women. Implementation of maternal or healthcare programs including the free maternal care should take the disadvantaged condition of rural women into account to ensure effectiveness of such programs in rural areas.

## Data Availability

Dataset analyzed during the current study are available at https://www.dhsprogram.com/data/dataset/Ghana_Standard-DHS_2003.cfm?flag=0 https://www.dhsprogram.com/data/dataset/Ghana_Standard-DHS_2008.cfm?flag=0 https://www.dhsprogram.com/data/dataset/Ghana_Standard-DHS_2014.cfm?flag=0.

## Abbreviations

DHS: Demographic Health Survey
GSS: Ghana Statistical Service
IPA: Innovations for Poverty Action
NHIA: National Health Insurance Authority
NHIS: National Health Insurance Service
UN: United Nations
